# A Wearable Assistive Device for Blind Pedestrians Using Real-Time Object Detection and Tactile Presentation

**DOI:** 10.3390/s22124537

**Published:** 2022-06-16

**Authors:** Junjie Shen, Yiwen Chen, Hideyuki Sawada

**Affiliations:** 1 Department of Pure and Applied Physics, Graduate School of Advanced Science and Engineering, Waseda University, Tokyo 169-8555, Japan; jj_shen@fuji.waseda.jp; 2 Department of Pure and Applied Physics, Faculty of Science and Engineering, Waseda University, Tokyo 169-8555, Japan; cyw8611@fuji.waseda.jp

**Keywords:** wearable device, object detection, SMA, tactile display, model compression, assistance for visually impaired people

## Abstract

Nowadays, improving the traffic safety of visually impaired people is a topic of widespread concern. To help avoid the risks and hazards of road traffic in their daily life, we propose a wearable device using object detection techniques and a novel tactile display made from shape-memory alloy (SMA) actuators. After detecting obstacles in real-time, the tactile display attached to a user’s hands presents different tactile sensations to show the position of the obstacles. To implement the computation-consuming object detection algorithm in a low-memory mobile device, we introduced a slimming compression method to reduce 90% of the redundant structures of the neural network. We also designed a particular driving circuit board that can efficiently drive the SMA-based tactile displays. In addition, we also conducted several experiments to verify our wearable assistive device’s performance. The results of the experiments showed that the subject was able to recognize the left or right position of a stationary obstacle with 96% accuracy and also successfully avoided collisions with moving obstacles by using the wearable assistive device.

## 1. Introduction

There are at least 2.2 billion people globally who have near or distant vision impairment, and 39 million of them are blind, according to the World Health Organization (WHO) [1]. However, because the methods for supporting their mobility are not very effective in many areas, it is still difficult for most visually impaired people to walk outdoors or travel without the help of an assistant. Compared with the non-disabled, blind people who have lost visual function become more dependent on other sensations, such as tactile sensation and auditory sensation. Therefore, different types of wearable devices using tactile sensations to transmit information about obstacles have been proposed to assist visually impaired people [2,3,4,5,6,7,8,9,10].

For example, Gemperle et al., developed a wearable tactile harness-vest display [6]. By combining six vibration motors, their wearable device can relay tactile messages, such as forward, back, left, right, speed up and slow down, to blind people. Although their wearable devices made a great breakthrough in improving the situation of blind people, we think that wearable devices should be able to detect obstacles in different environments in real-time. The outdoor environment can be more complex than a carefully designed test field. In this paper, we tried to determine how to deal with objects that appear suddenly in a new environment instead of a designed maze.

Wagner et al. proposed an impressive tactile display that presents different tactile sensations using commercial RC servo motors. Because the aim is to assist visually impaired people in their daily life, the devices are required to be compact and portable. However, to achieve higher performance, the hardware requirements of the wearable device usually increase. These requirements result in the device becoming unportable and expensive, which is not conducive to promoting and popularizing their use among blind people.

In our study, we proposed a wearable assistive device that can not only detect real-time obstacles, but can also help visually impaired people avoid collisions by using portable and small tactile displays. To realize high-performance real-time object detection while maintaining the portability of the device, we compressed the model of a high-performance object detection algorithm called YOLOV3 (You Only Look Once Version 3) [11] and successfully deployed the compressed model on a mobile device. We also designed a compact tactile actuator based on a 0.75 mm diameter SMA wire, which is small and can vibrate quietly. We also designed a driving circuit to reduce the space occupancy further and ensure stability to drive these actuators efficiently.

## 2. Materials and Methods

### 2.1. Wearable Device for Blind Pedestrians

**Figure 1:**
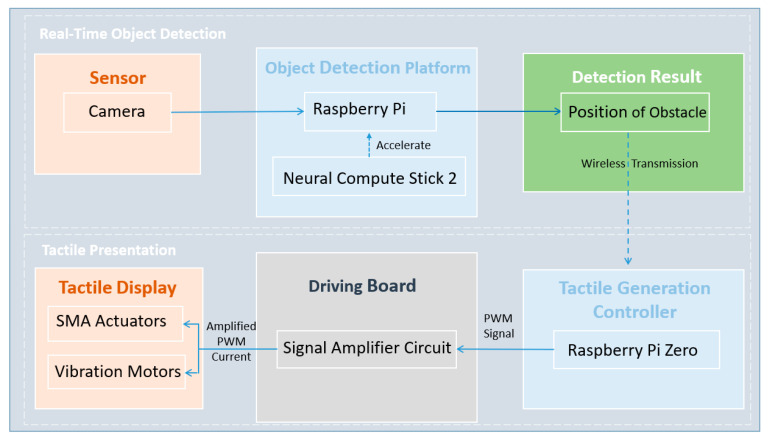
Constructure of the wearable device.

Our wearable device for assisting blind pedestrians mainly contains two sub-devices: real-time object detection and information presentation through tactile sensation. The relationship between each part of the sub-devices is shown in Figure 1. By deploying the compressed YOLOV3 model on Raspberry Pi 3B+ accelerated by Neural Compute Stick 2 (NCS2), the primary function of the real-time object detection device is to detect pedestrians in front of a blind user in real-time. Then, the detection results are transmitted wirelessly to the tactile presentation device based on Socket Communication. According to the received information, the tactile presentation device provides notifications to users using different tactile sensations and by controlling the tactile actuators.

### 2.2. Real-Time Object Detection Using Compressed YOLOV3 Model and NCS2

**Figure 2:**
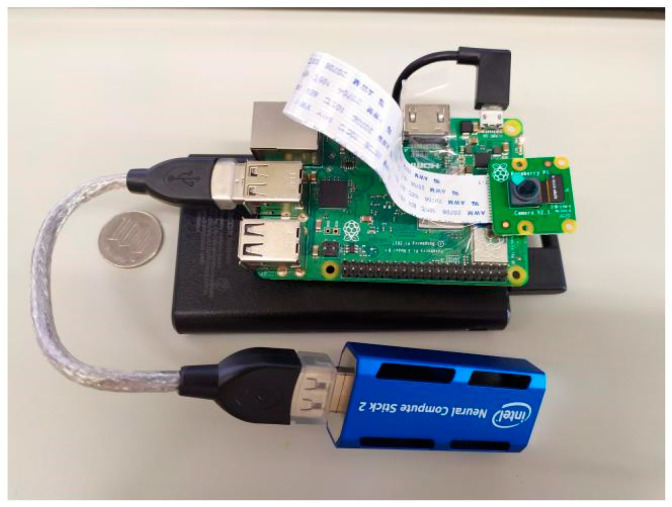
Constructure of real-time detection sub-device.

The main components of real-time object detection include a Raspberry Pi 3B+, NCS2, and Raspberry Pi Camera Module 2 with 8 million pixels. To provide the Raspberry Pi 3B+ with a stable power supply, we chose a 5000 mAh mobile power supply, which is enough to keep the device running for more than 3 h. An image of the entire real-time object detection device is shown in Figure 2.

Even though the Raspberry Pi+ is lightweight and has low power consumption, a high-performance object detection algorithm such as YOLOV3, which has more than sixty million parameters, cannot be directly deployed due to its insufficient computing power. To realize high-performance object detection while keeping the device’s compactness, we mainly used two methods, that is, YOLOV3 model compression and the acceleration of NCS2.

#### 2.2.1. YOLOV3 Model Compression

First, a model compression method was used to remove the redundant structures of the neural networks. We introduced a layer and channel pruning method to compress the YOLOV3 model. After the compression, the size of the YOLOV3 model decreased to 5% of its original size, leading to a significant increase in the speed of model inference. This slimming compression method contains four stages: initial training, sparsity training, channel pruning and a fine-tuning stage [12]. The data and results of each step are given in Section 3.

#### 2.2.2. Accelerate Compressed Model by NCS2

The second method was used to accelerate the device by NCS2. Because of the low price and compact size requirements, mobile devices generally do not load GPUs. Therefore, some large-size image processing or object recognition algorithms cannot perform with high accuracy on mobile devices. NCS2 can solve this problem by providing edge computation to optimize the compressed model and accelerate the calculation speed. The details of using NCS2 are given in Section 3.

### 2.3. Tactile Presentation Device Using SMA and Vibration Motors

**Figure 3:**
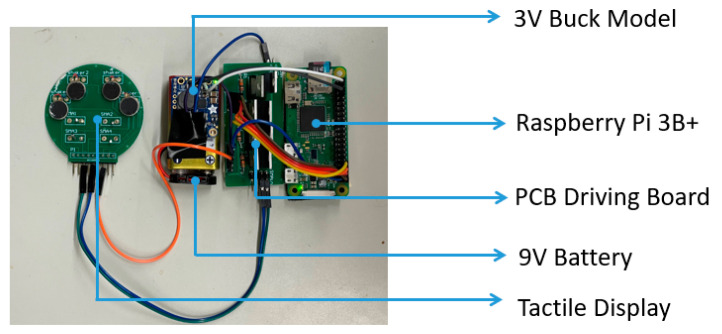
The tactile presentation device.

The tactile presentation device contains a Raspberry Pi Zero, a 9 V battery as the power supply, a 3 V buck module, two driving boards, and a tactile display consisting of SMA actuators. Raspberry Pi Zero is regarded as the core controller that receives the obstacle information from the real-time object detection wirelessly, and controls the general-purpose input/output (GPIO) to output pulse width modulation (PWM) current with different frequencies and duty ratios. After passing the driving board, the PWM current outputted by Raspberry Pi Zero is amplified and then used to drive the tactile display. The amplified PWM with different frequencies and duty ratios, controls the tactile actuators to present different tactile sensations. The tactile presentation device is shown in Figure 3.

#### 2.3.1. Tactile Display

**Figure 4:**
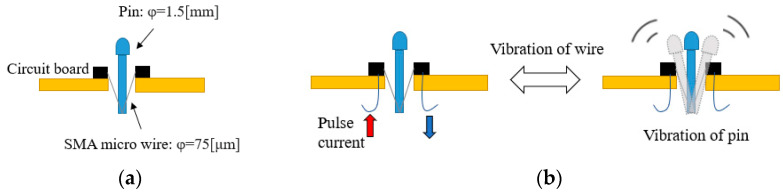
Structure of SMA actuators. (**a**) Structure of pin-type tactile actuator driven by SMA wire. (**b**) Stimuli presented by vibrating pin.

The tactile display is designed to transmit obstacle information to a blind person by presenting different tactile sensations. To enrich the types of tactile sensations presented by the tactile display, we combined two types of tactile actuators in the tactile display: shape-memory alloy (SMA) actuators and vibration motors. Both of them can be electrically driven by periodic signals. SMA actuators are composed of a metal pin and a 5 mm long SMA wire with a diameter of 0.075 mm [13]. The structure is shown in Figure 4.

SMA is a metal that can change its shape when the temperature changes. When the temperature rises to the transformation temperature, the length of the SMA wire shrinks to 5% of the original length [14]. Then, this deformation of the shrinkage can be amplified by the metal pin, as shown in Figure 4. The PWN current is used to drive the SMA actuators of our device.

**Figure 5:**
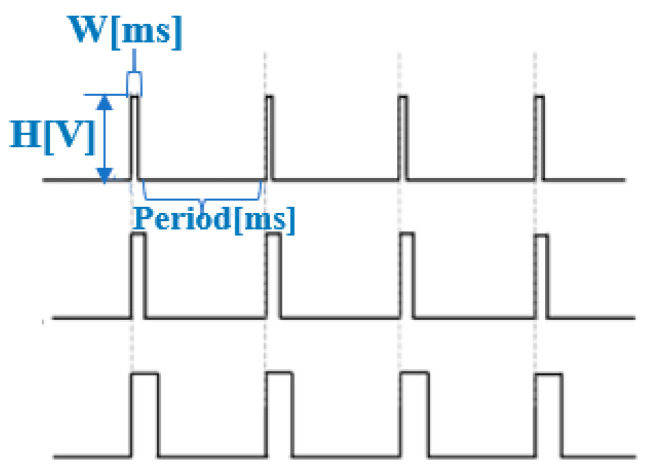
Waveforms of PWM.

The PWM wave has two states in one period: ON and OFF. The duty cycle reflects the proportion of the duration of the PWM cycle in the ON state. A high duty cycle corresponds to high power because the time of the ON state is longer than the time of the OFF state. When the PWM wave is in the ON state, current flows through the SMA actuators, and the current generates energy to heat the SMA wire, causing it to shrink. In the OFF state, the SMA wire cools down by radiating heat into the air, and return to its original length. The waveforms of the PWM are shown in Figure 5.

When the amplitude of PWM is expressed as H (V) and the time of ON states is expressed as W (ms), W*H, which is equivalent to the electrical energy, represents the current energy. Therefore, we used Raspberry Pi zero to control the frequency and duty cycle of the PWM wave to make the SMA actuators generate vibrations of different frequencies and intensities. Vibrations with different frequencies are perceived as different tactile sensations by the users.

The vibration motor is also controlled by the PWM wave. Compared with SMA actuators, the vibration of the vibration motor is stronger and more easily perceived by a user. Since the vibration frequency of the vibration motor cannot be changed, the type of produced tactile sensation is a particular one. Since the SMA actuators and the vibration motors have their advantages, we have combined them to design two different tactile displays, tactile display 1 and tactile display 2.

**Figure 6:**
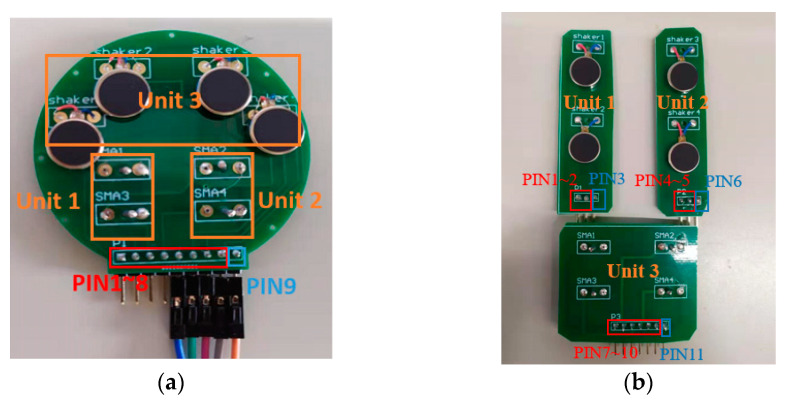
Tactile displays. (**a**) Tactile display 1. (**b**) Tactile display 2.

We attached four vibration motors and four SMA actuators to tactile display 1, as shown in Figure 6a. The input of each actuator has its pin assigned as PIN1~8. The output of each actuator was connected together and assigned as PIN 9.

Tactile display 2 was designed as a distributed architecture, consisting of unit 1, unit 2 and unit 3 as shown in Figure 6b. Two sets of vibration motors were attached to two different displays on the top, and four SMA actuators were attached to the display below. The input of each tactile generator also had its pin assigned as PIN1~3, PIN4~5 and PIN7~10, while the output was assigned as PIN3, PIN6 and PIN11, respectively.

#### 2.3.2. Current Amplifier Circuit as Driving Board

We found that a 5 mm long SMA wire with a diameter of 0.075 mm can generate obvious deformation powered by a 0.8 A current, at least. However, Raspberry Pi can only output logical signals (3.3 V as high and 0 V as low), and the output current value is only about 80 mA.

**Figure 7:**
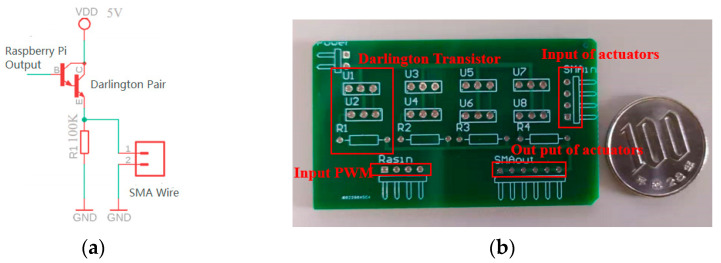
Current amplifier circuit. (**a**) Darlington transistor current amplifier. (**b**) Outlook of driving board.

To amplify the output current, we designed a current amplifier circuit as a driving board to connect with the GPIO of Raspberry Pi. The driving board was designed based on the Darlington transistor current amplifier, which is composed of two ordinary bipolar transistors, as shown in Figure 7a. In our wearable device, multiple tactile actuators are independently controlled to present tactile sensation at the same time. To make the control efficient, we designed the driving board as a multiple I/O current amplifier circuit, as shown in Figure 7b.

## 3. Results

### 3.1. YOLOV3 Model Compression

#### 3.1.1. Initial Training

**Figure 8:**
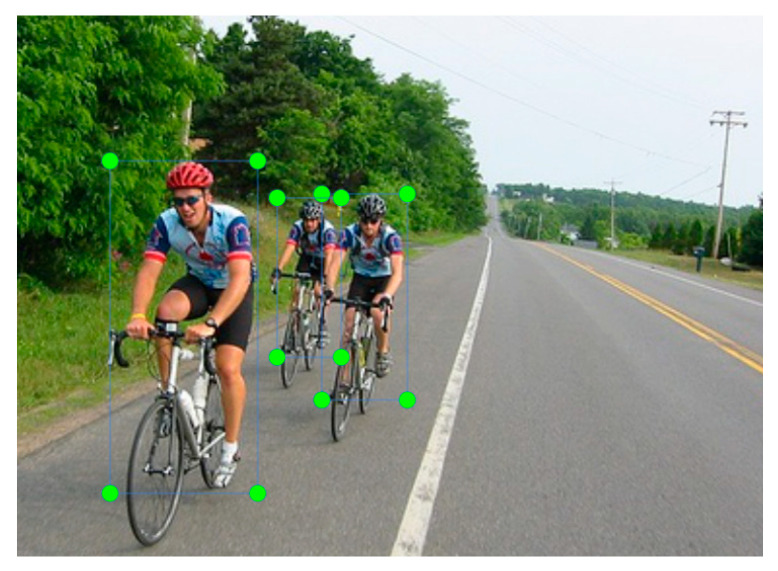
An example of images used for training the model.

We sampled 4193 images of pedestrians from the COCO dataset. These images were used with annotations for training the YOLOV3 model at this stage. Among the 4193 images, we used 3353 images for the training set and 839 images for the verification dataset. An example of the tagged images we used to train the model is shown in Figure 8.

In our experiment, the YOLOV3 was trained for 100, 300, and 200 epochs in each training stage, respectively, by using the stochastic gradient descent (SGD) optimization method. The initial learning rate was 0.001, and the batch size was 64. We also recorded the precision, recall, and loss value to measure the performance of YOLOV3 during the training process. The loss value is a measurement of how well the model is able to predict the expected outcome. The smaller the loss value becomes, the closer the model’s predicted result is to the actual value. The other two metrics can be defined according to the confusion matrix shown in Table 1.

The precision metric indicates the proportion of samples that are predicted to be positive samples that are truly positive samples. It is used to describe the detection accuracy of the object detection algorithm, given by

(1) $$\mathrm{Pr}\mathrm{ecision}=\frac{\mathrm{TP}}{\mathrm{TP}+\mathrm{FP}}$$

The recall metric indicates the number of positive objects predicted as positive images in all labeled images. It is used to describe the prediction ability, given by

(2) Recall=TPTP+FN

**Figure 9:**
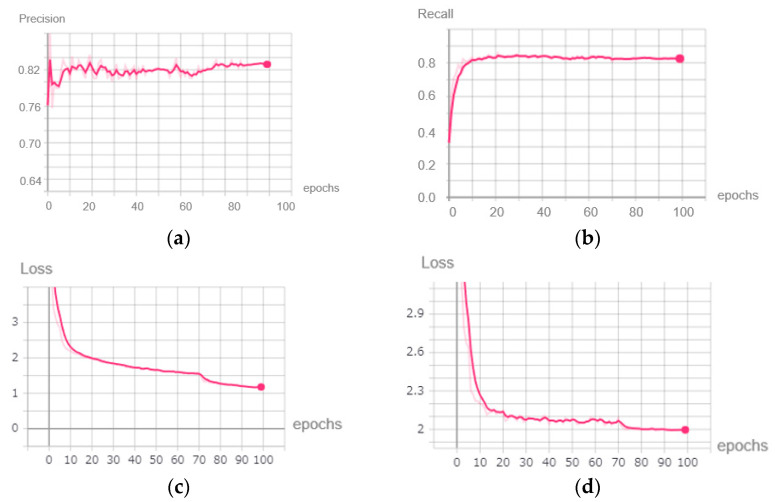
Metrics of the initial training stage. (**a**) Precision. (**b**) Recall. (**c**) Loss value of the training dataset. (**d**) Loss value of the testing dataset.

The precision, recall curve, and loss value of the training and testing dataset during the initial training are shown in Figure 9.

We concluded that as the training progressed, the precision and recall of the YOLOV3 model continued to increase and reached above 0.82 at the end of the initial training, while the loss value of the model dropped from 6.903 to around 1.183 in the training dataset and from 4.984 to 1.996 in the testing dataset. Therefore, in this initial training stage, the YOLOV3 model began to have some ability to detect objects. The model at this time had good performance on a computer with GPU support. However, it was still difficult to deploy it on mobile devices because the memory of the mobile device was too low to run millions of parameters.

#### 3.1.2. Sparsity Training

From Figure 9, it can be noted that the YOLOV3 model became over-fitted at the end of the initial training stage. The over-fitting problem can be solved by adding L1 regularization to the loss function and re-training the YOLOV3 model. The loss function in the sparsity training stage was given by

(3) $$\mathrm{Loss}=\sum_{\left(x,y\right)}l\left(f\left(x,W\right),y\right)+\lambda \sum_{\gamma \epsilon \Gamma }\left|\gamma \right|,$$

where x and y were the training input and target value, respectively. W denoted the trainable weights of the neural networks. The first part of the loss function represents the loss function of YOLOV3, while the second part is known as L1 normalization, which is regarded as a method to solve the overfitting problem. γ is the scaling factor of each channel of YOLOV3, and λ balances two parts of the loss unction.

**Figure 10:**
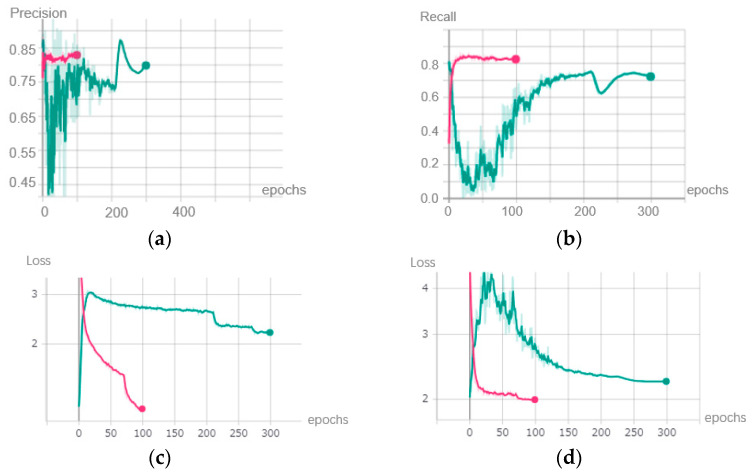
Metrics of the sparsity training stage. (**a**) Precision. (**b**) Recall. (**c**) Loss value of training dataset. (**d**) Loss value of testing dataset.

We used this new loss function to train the YOLOV3 model again. Not only can the overfitting problem be solved, but the distribution of the weights can also be adjusted. The precision, recall, and loss value of the training and testing dataset during the sparsity training is shown in the green curve of Figure 10.

The green lines in Figure 10 represent the plots of the sparsity training. Compared with the purple lines, which refer to the initial training, the precision and recall decreased greatly at the beginning because L1 regularization was added. After the 50th epoch, the metrics of the model began to increase again, and the loss value of the training dataset and testing dataset also began to decrease.

**Figure 11:**
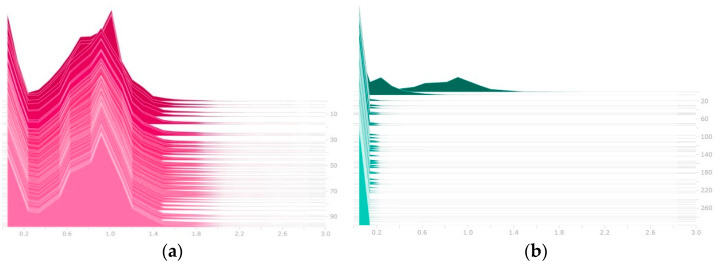
Distribution of trainable weight. (**a**) Trainable weight before sparsity training. (**b**) Trainable weight after sparsity training.

Although the accuracy metrics of the model were constantly oscillating because L1 regularization was added to the loss function, most of the model’s trainable parameters became close to 0. The distribution of trainable parameters before and after sparsity training is shown in Figure 11.

#### 3.1.3. Layer Pruning and Channel Pruning

After the sparsity training, many trainable parameters were close to 0 so that a threshold could be set to prune the whole network. More specifically, if the value of the trainable parameters was lower than the threshold, it would be set to 0.

The results of pruning are shown in Table 2. The number of parameters of the YOLOV3 model decreased from around 61 million to 800,000. However, precision and recall suffered heavy setbacks. The value of precision dropped from 0.79 to 0.39 and recall also dropped from 0.72 to 0.6781.

Although the YOLOV3 model was significantly compressed, the accuracy of the model also decreased severely. The YOLOV3 model could barely recognize any object at this time. To recover the loss of accuracy, we fine-tuned the model in the next stage.

#### 3.1.4. Fine-Tuning

After layer pruning and channel pruning, the number of parameters of YOLOV3 decreased, which significantly reduced the amount of computation on the mobile device. However, the accuracy of the YOLOV3 also became notably poor because a large number of training parameters were zeroed. To improve the accuracy again, we fine-tuned the pruned YOLOV3 model in this stage.

**Figure 12:**
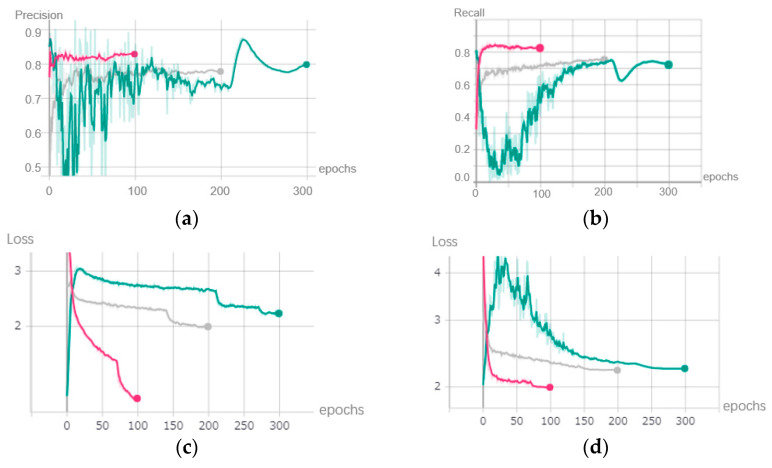
Metrics after fine-tune stage. (**a**) Precision. (**b**) Recall. (**c**) Loss value of training dataset. (**d**) Loss value of testing dataset.

In the fine-tuning stage, we retrained the pruned model for 100 epochs. The grey lines in Figure 12 show the trend in the metrics in this stage. The accuracy metrics such as precision and recall increased smoothly, and the loss value decreased constantly. The detection accuracy of the model recovered gradually.

#### 3.1.5. Summary of Compression

We used the slimming algorithm to compress the YOLOV3 model described in Section 3.1. The number of parameters of the model dropped from 60 million to 1.3 million, which reduced the size of the weight file from 236 MB to 3.1 MB. A smaller weight file means less computation, which makes it possible for us to deploy the YOLOV3 model on a low-memory mobile device.

### 3.2. Accelerating Compressed YOLOV3 Model Using NCS2

**Figure 13:**
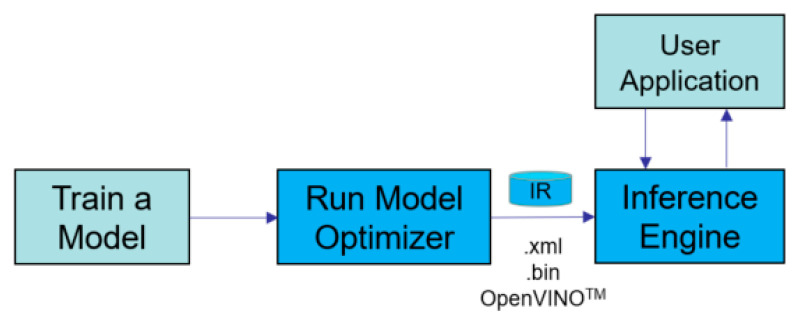
Step for utilizing OpenVINO to accelerate model.

By employing the OpenVINO toolkit, we utilized NCS2 to optimize the YOLOV3 model and accelerate the inference speed. The OpenVINO toolkit is a comprehensive toolkit for quickly developing applications and solutions [15]. The steps for using OpenVINO to optimize and accelerate the model are shown in Figure 13.

The Model Optimizer tool provided by the OpenVINO was firstly used to convert the YOLOV3 model into an IR format, which is a special format supported by OpenVINO. Because different deep learning frameworks such as Tensorflow and Caffe have their own format for the weight file, converting these different weight files into the same IR format is regarded as a good solution to solve the compatibility problem. After obtaining the model in IR format, we used the Inference Engine tool to quickly build our application.

With the support of NCS2, the inference speed of the YOLOV3 model was greatly improved in Raspberry Pi. The performance of Raspberry Pi 3B+ is shown in Table 3. Because of a large amount of computation, the YOLOV3 model cannot be deployed on the Raspberry 3B+ directly. However, with the acceleration of NCS2, Raspberry Pi 3B+ can run the YOLOV3 model with an inference time of 0.5 s.

The compressed YOLOV3 model can be deployed in Raspberry Pi 3B+ directly but the inference time is more than 3 s. After being accelerated by NCS2, the inference time can be shortened to 0.3 s.

### 3.3. Experiment

For verification, we conducted two experiments.

#### 3.3.1. Experiments for the Tactile Display

**Figure 14:**
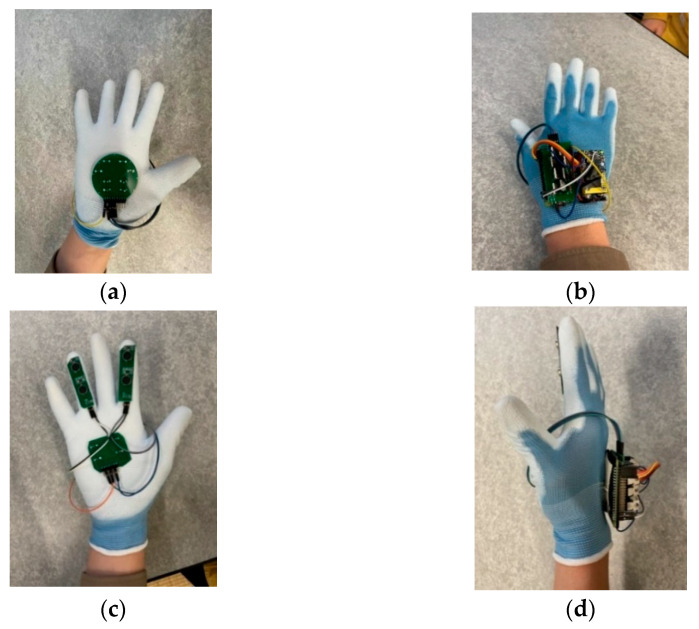
Two kinds of tactile display attached to the glove. (**a**) Tactile display 1 attached to the palm of the gloves. (**b**) Control circuits attached to back of gloves. (**c**) Tactile display 2 attached to the palm of the gloves. (**d**) Control circuits attached to back of gloves.

Firstly, to verify the performance of the tactile display, we conducted 120 sets of experiments on three male subjects aged 25, 24, and 20. The heights of the subjects were 185, 175, and 176 cm. All subjects were blindfolded with an opaque black cloth. Sixty sets of experiments were conducted in the morning (9:00–11:00 a.m.), and 60 sets of experiments were conducted in the afternoon (14:00–16:00 p.m.). Two kinds of tactile displays, as shown in Figure 6, were attached to the palm of the gloves while other control circuits for tactile presentation were attached to the back of gloves, as shown in Figure 14.

**Figure 15:**
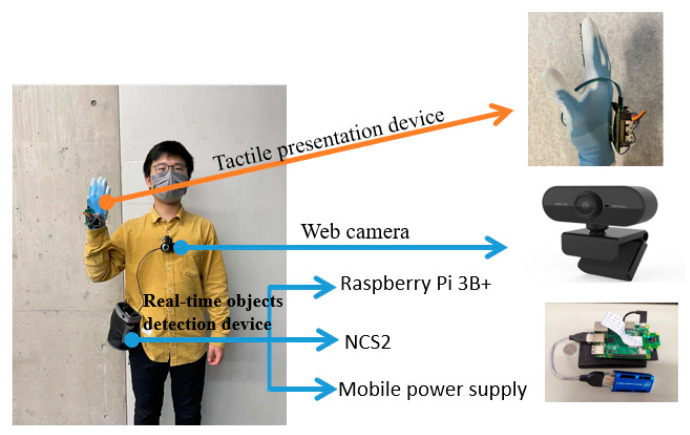
Subject wearing devices.

Figure 15 shows a subject wearing the whole assistive wearable device. The calculation part of the real-time object detection, which contains the Raspberry Pi 3B+, NCS2 and mobile power supply, was put into a waist pack on a belt, while the web camera was attached to the subject’s chest to obtain first sight vision. The real-time object detection sent the positions of the nearest object to the tactile presentation device wirelessly. Based on the detected positions, the Raspberry Pi Zero outputted the PWM signal, and controlled the tactile actuators on the glove to present different tactile patterns. The relationships between a detected object and a tactile pattern are shown in Table 4 and Table 5.

**Figure 16:**
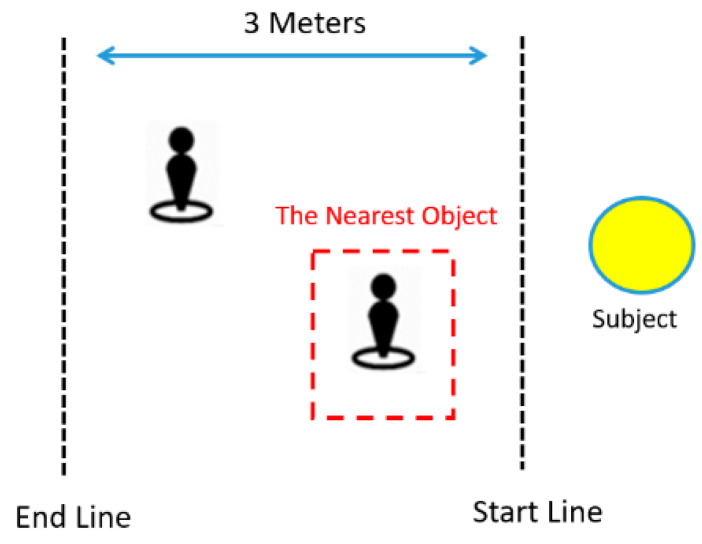
Journey with one or two pedestrians.

In the experiments, we set a 3 m journey and used one or two pedestrians as obstacles, as shown in Figure 16. The pedestrians stood on the right or left of the blind subject, while the subject judged the positions of the nearest pedestrians by wearing the tactile glove.

To record the effectiveness of the tactile display, we recorded the subject’s judgment (go left or go right) when facing the nearest pedestrian, as shown in Table 6. According to the results, the tactile sensation produced by our tactile display was felt clearly in most situations.

#### 3.3.2. Experiments for the Wearable Device

After verifying the performance of the tactile display, we conducted more experiments to test the performance of the wearable device. In this experiment, we invited three people as subjects and conducted 45 experiments in three situations.

In the first situation, only one pedestrian stood in front of the subject at a distance of 1.5 m while in the second situation, two pedestrians stood in front of the subject. One was 1.5 m away from the subject, and the other was 2.5 m away from the subject. In the third situation, the subject and two pedestrians walked towards each other. The distance between the two pedestrians was one meter, and the initial distance between the subject and the nearer pedestrian was about two meters.

In the experiments, the subject with our wearable device tried to navigate the journey without any collisions. To prove the effectiveness of the wearable device, we recorded the number of collisions during the whole journey, as shown in Table 7.

## 4. Discussion

From the results shown in Table 7, the subject was able to successfully avoid collisions many times in a simple environments such as Situation 1 and Situation 2 by using our wearable device. Especially in Situation 1, the user successfully avoided all obstacles in 45 experiments. In Situation 2, the first subject avoided all collision successfully. The second subject had one collision on the left and one collision on the right, while the third subject only had one collision on the left.

However, when the situation became more complex such as in Situation 3, the number of collisions became higher. The first and second subjects both had two collisions on the right and one on the left. The third subject had two collisions on the left.

The reason was that the obstacle and the subject were moving towards each other, and even if our wearable device detected the obstacle, the time available for the subject to reflect was very limited. However, in the real world, blind people are usually noticed by moving pedestrians in advance, and these moving pedestrians will proactively avoid collision with the subject. This action can further reduce the number of collisions.

After the experiments, we interviewed three subjects and all of them confirmed that the wearable device was lightweight. Because the real-time object detection system is deployed on the mobile device, the device is compact and easy to wear. Two subjects said it was easy to get confused in the initial period of using our wearable device, but they also confirmed that they were able to use it proficiently after some practice. In terms of performance, the wearable device had a good performance in simple environments such as Situation 1 and Situation 2. However, in a complex environment like Situation 3, the performance of the wearable device still needs to be improved.

With the development of current research, more object detection algorithms that have better performance than YOLOV3 have been proposed, such as TridentNet, and NAS-FPN [16,17]. They can detect obstacles faster and more accurately. Using other object detection algorithms that have better performance than YOLOV3 can be regarded as a means of improving the performance of the wearable assistive device.

## 5. Conclusions

In this study, we built a wearable device for blind pedestrians to improve their mobility safety. This wearable device contains two parts: real-time object detection and information presentation through tactile sensation. Because we care about the compactness of the wearable device, all the programming and computation was deployed on a mobile device.

Regarding the real-time object detection device, running the complex YOLOV3 model directly on the mobile device is very difficult because of the memory limitation. To solve this problem, we compressed the YOLOV3 model by 95%, which resulted in faster inference speed on a mobile device.

Regarding the tactile presentation device, we used SMA and vibration motors to represent different tactile sensations. To drive these micro-vibration actuators, we also designed a PCB driving board, while trying to keep the device’s compactness.

According to our experiments, the subjects were able to navigate the journey without collision in most situations. In addition, the combination of the shape memory-alloy tactile actuators and the vibration motor on the tactile display can be regarded as a good way to generate different tactile sensations and help blind people to avoid obstacles in the traffic.

## Figures and Tables

**Table 1:** Confusion matrix.

		True Value	
		True	False
Prediction	True	True Positive (TP) Correct	False Positive (FP) Type 1 Error
	False	False Negative (FN) Type 2 Error	True Negative (TN) Correct

**Table 2:** Metrics before and after sparsity training.

Metric	Before Pruning	After Pruning
Parameters	61,523,734	809,434
Precision	0.7988	0.3964
Recall	0.7200	0.6781

**Table 3:** Performance of YOLOV3 model and pruned-YOLOV3 model.

Model	Parameters	Size	Inference Time	Inference Time (NCS2)
YOLOV3	62,573,334	236 MB	unknown ^1^	0.5 s
Compressed YOLOV3	809,434	3.1 MB	3.43 s	0.3 s

^1^ The YOLOV3 model cannot be deployed on Raspberry Pi directly because of the large memory consumption.

**Table 4:** Relationships between a detected object and a tactile pattern using tactile display 1.

Object Position	Tactile Pattern
Right Side	Actuators on Unit 1 shake in 10 Hz
Left Side	Actuators on Unit 2 shake in 10 Hz
No Object	Actuators on Unit 3 shake in 2 Hz

**Table 5:** Relationships between a detected object and a tactile pattern using tactile display 2.

Object Position	Tactile Pattern
Right Side	Actuators on Unit 1 shake in 2 Hz
Left Side	Actuators on Unit 2 shake in 2 Hz
No Object	Actuators on Unit 3 shake in 10 Hz

**Table 6:** Accuracy of identifying and judging the position of obstacles.

Position of Obstacle	Judgment	
	Left	Right
entry 1	97% (Success)	3%
entry 2	4%	96% (Success)

**Table 7:** Number of collisions in each situation.

	Number of Experiments	Collisions on the Left	Collisions on the Right	Total Number of Collisions
Situation 1	45	0	0	0
Situation 2	45	2	1	3
Situation 3	45	4	4	8

## Data Availability

The data presented in this study are available on request from the corresponding author.
